# What Motivates Internet Users to Search for Asperger Syndrome and Autism on Google?

**DOI:** 10.3390/ijerph17249386

**Published:** 2020-12-15

**Authors:** Raúl Tárraga-Mínguez, Irene Gómez-Marí, Pilar Sanz-Cervera

**Affiliations:** Department of Education and School Management, Faculty of Teacher Training, University of Valencia, 46022 Valencia, Spain; irene.gomez@uv.es (I.G.-M.); pilar.sanz-cervera@uv.es (P.S.-C.)

**Keywords:** Asperger, autism, Google trends, internet users’ behavior, MyNews, social campaigns, Trendinalia

## Abstract

Social campaigns are carried out to promote autism spectrum disorder (ASD) awareness, normalization, and visibility. The internet helps to shape perceptions of Asperger syndrome and autism. In fact, these campaigns often coincide with the increase in searches for both diagnoses on Google. We have two study objectives: to use Google Trends to identify the annual time points from 2015 to 2019 with the highest Google search traffic in Spain for the terms “autism” and “Asperger”, and to identify news and trending topics related to ASD that took place during the weeks with the highest number of Google searches for these terms. Google Trend, MyNews and Trendinalia were used to analyze the volume of searches and trending topics related to ASD. As a result, social marketing campaigns, social networks and the publication of news items act as powerful voices that can provide a realistic or sensationalist picture of the disorder. For this reason, we concluded that campaigns play an important role in the normalization of ASD, and that it is important for organizations concerned with the visibility and social inclusion of people with ASD to check the way ASD is portrayed through the internet, media, and social networks.

## 1. Introduction

Nowadays, the content of internet searches is an indicator of the issues that concern or interest to the public. It is increasingly common that, when people are struck by a piece of news in the press, on TV, or in a Twitter conversation, they google to satisfy their queries. Thus, Google search patterns become a valuable indicator that provides us with frequent information requested on internet by users at all times. This information is valuable in areas such as the defense of the rights of people with disabilities, where one of the historical aspirations has been to achieve high levels of social visibility.

In this paper, we analyze when the Google search peaks for the terms “autism” and “Asperger” have occurred in Spain from 2015 to 2019. In addition, we try to analyze in which extent news in the media and/or conversations on Twitter are related to these search peaks. We believe that the results of this study can help to know which social events and/or awareness campaigns attract the most attention from internet users. It can also help to identify what kinds of events are contributing to perpetuate stigmatization and false myths about people with autism spectrum disorder (ASD). This information can be valuable to the agents responsible for designing and implementing awareness campaigns on ASD.

### 1.1. From Asperger’s to Autism Spectrum Disorder: Modifications in Diagnostic Criteria

The classification of some disorders underwent important changes with the 2013 publication of the fifth edition of the “Diagnostic and Statistical Manual of Mental Disorders” (American Psychiatric Association, (APA)). In the case of ASD and Asperger syndrome, the main modifications in the new categorization are summarized in Table 1.

Asperger syndrome, described by the DSM-IV-TR [1] as one of the generalized developmental disorders and characterized by a severe and persistent alteration in social interaction with restrictive and repetitive patterns of behavior, interests, and activities, is now diagnosed as ASD (normally “level 1”, because the symptoms of the disorder are not usually manifested in a significant way: there are neither significant language delays nor significant alterations in cognitive development).

The diagnostic criteria for ASD are also considerably modified. The DSM-5 [2] describes it as a neurodevelopmental disorder characterized by persistent deficits in social communication and social interaction in several contexts, along with restrictive and repetitive patterns of behavior, interests, or activities, in addition to presenting alterations in sensory input. Depending on the degree of help each individual needs, the level of autonomy varies from 1 to 3, with 1 being the least affected.

### 1.2. Social Impact of Diagnostic Label and Internet Search Behavior

Several studies [3,4,5] have analyzed whether these changes in the name of Asperger syndrome might lead to an increase in stigmatization and prejudices about people with this diagnosis. However, other authors [6,7] conclude that both the Asperger’s and ASD diagnoses receive positive treatment attitudes, with no significant differences between them.

The internet helps to shape perceptions—positive or negative—about the ASD and Asperger syndrome labels [8,9]. It is increasingly common for people (especially those with limited resources) to make use of different websites to search for and satisfy their queries on any subject [10]. Platforms such as Facebook, Twitter, and Google have become absolute spreaders of information about the autism and Asperger’s diagnoses [11,12,13].

In some cases, the content offered on the websites corresponds to myths, hoaxes, fallacies, sensationalism, creating controversy, etc., as in the following cases: false myths about vaccines [14]; misleading cures for the disorder [15]; attributing death to the diagnosis itself [8]; or the controversy and curiosity generated by the “Greta phenomenon” [16,17,18]. In other cases, the same platforms can act as standardization, awareness, and inclusion tools. Quality—grounded—and positive information, in addition to propagating initiatives and awareness campaigns, can break with the unfounded prejudices created by false myths [13,17]. Studies guarantee that positive media coverage of influential figures with certain diagnoses, in this particular case, autism or Asperger syndrome, can have a unique ability to enhance visibility and normalization. Therefore, the media can also play a role in destigmatization, providing society with education and realistic descriptions of diagnoses and debunking falsehoods and myths.

When addressing this issue, it is necessary to take into account that the analysis of the images that the media and the internet show about ASD and Asperger syndrome are not neutral [19]. Several studies have shown biases in the treatment of ASD (and other disabilities) by mass media. For example, a study conducted in United Kingdom [20] compared the portrayal of disability by the same media in the years 2004 and 2005 (prior to the economic crisis), and 2010 and 2011 (years marked by adjustments in public spending due to the economic crisis). This study evidenced that during the economic crisis period the media coverage of disabilities had a much less empathic treatment than in the pre-crisis period. There was an evident improvement of news reporting cases of fraud related to public aid to families of people with disabilities and there was also an increase in pejorative language towards people with disabilities. Other studies have focused on analysing how the media can contribute to perpetuate or reduce the stigmatization attached to autism through the publication of news that constantly influences the same approaches, thus generating a framework for readers, influencing their perception of ASD [21,22,23]. Therefore, the description of the prototype of a person with ASD or Asperger’s is a construction produced by specific agents (such as mass media) according to certain interests. Therefore, when analysing the picture projected regarding ASD and Asperger’s, we should consider which specific agent elaborates this projection and what interests it has.

### 1.3. Visibility and Normalization: Towards Social Inclusion

Periodically, initiatives are carried out to promote awareness, normalization, and visibility and make known the reality of people with diagnoses of ASD or Asperger syndrome, and these initiatives coincide with increases in searches about autism or Asperger’s. Specifically, in this section we mention four of them: the promotion of world days [24]; campaigns on social networks such as Twitter [25] or Facebook [16]; campaigns carried out by organizations such as the National Alliance of Mental Illness, called *CureStigma* [17]; televised broadcasts about autism or where ASD plays a remarkable role [26].

Some authors [27] contend that campaigns have a very positive effect on raising awareness and disseminating information, especially among young people. Therefore, health campaigns should use these platforms to reach a wider audience. Regarding the content of websites, it is interesting to analyze the way the internet offers its users the specific content [8,9,28]. Websites can shape ideas about inclusion and disseminate rigorous information or, on the contrary, lack basic content about the diagnosis and its intervention mechanisms [29,30] from searches and information (based on supply and demand, as we will see in the following section).

Thus, the media and the internet are tools with great potential to influence the social construction of disability and they also serve to raise awareness of the existence of environmental barriers that must be eliminated, to make easier the configuration of more favourable attitudes and facilitate the social inclusion of people with disabilities. However, they can achieve the opposite purpose: to ignore the existence of barriers and therefore contribute to perpetuate or even enlarge them.

### 1.4. Infodemiology

In the internet age, information is just a click away. The analysis of searches has become a useful method to understand and explain human behavior [31] and discover the population’s level of awareness about different conditions such as autism [32]. Understanding search trends and search behavior, as well as the frequency and location of searches in time and space, can be effective for developing public health policies [25,28,33].

Infodemiology deals with this accurately. Additionally, infodemiology evaluates the content of internet information related to health [31] in order to determine its quality indexes [28]. It has two different and complementary aspects: health information demand and health information supply [33]. Some authors [28] conclude that when the supply of researchers—studies—considers the demand of internet users—the most frequent queries and searches—then, the line of inquiry draws closer to the health needs of the population [34]. For this purpose, some studies have used the Google Trends tool [16,26,31].

In infodemiology research, it has been contended [24,25] that the highest search peaks for autism or Asperger syndrome are usually cyclical. In other words, every year searches tend to skyrocket on 18 February (International Asperger’s Day (IAD)) and on 2 April (World Autism Awareness Day (WAAD)), or even during all of Autism Awareness Month [26]. In any case, the volume of internet searches can also be influenced by specific events, such as TV broadcasts related to autism in prime time.

Some authors [26] specified that the increase in searches for ASD on Google has occurred every month of April from 2004 to 2014, except in 2005. Throughout the month of March (for ten years), according to the authors, there is an increase in searches until the beginning of April (WAAD is 2 April), followed by a decrease in the second half of April and May. The peak in searches throughout this decade was in April 2008, possibly thanks to the initiative that had been organized at the end of the previous year, in 2007, announcing that, from then on, autism day would be 2 April. Another increase occurs in September. Going back to school immerses many teachers in the search for techniques, interventions, and advice on how to interact and deal with students with autism or Asperger syndrome. Others [16] discovered a peak in searches for “autism and vaccines” in 2012, in April, International Autism Awareness Month. In that study, the researchers show how social networks provide a loudspeaker for anti-vaccine campaigners who associate vaccination with the diagnosis of autism or Asperger’s: the creation of walls, posts, and sites on Twitter and Facebook contributes to the spread of rumors, myths, and, ultimately, misinformation in this specific case.

Some researchers [17] point out the upward trend in the most recent internet search related to Asperger’s. It arises during the United Nations Climate Summit week (coinciding with the appearance in the media of Greta Thunberg, a young activist with Asperger syndrome) and runs until the end of the year when her distancing from the media caused internet users to take an interest in her, increasing the volume of searches for the terms “Asperger” and “autism”. One study [25] shows peaks coinciding with WAAD, not only in searches, but also in trending topics (TT) produced by Twitter users.

It is ensured that the increase in searches in different months other than April may be related to the Greta phenomenon [16,17] and to the multiple television formats that are recently reporting on autism and Asperger’s or include main characters with these diagnoses (for example, according to [35], Dr. Murphy in the hit series “The Good Doctor”). In any case, knowing what motivates internet users to search the internet for information about autism or Asperger’s can be a beneficial way to contrast and evaluate the information that shapes their perceptions of the disorder [28,31].

Knowing this information can be useful: (a) for associations that organize and carry out awareness campaigns, since the results obtained will provide them with information about the effect of said campaigns, in addition to knowing which events have an impact on Google searches; (b) for those in charge of elaborating public policies related to inclusion, since through this information they can find out which specific aspects interest the population the most; and (c) for teacher trainers and professionals who work with children with ASD, who are agents responsible for generating attitudes towards these children, so it is key to know what is included in the network, with the aim of banishing possible myths and/or false beliefs.

Most of the research carried out in this field at an international level has been conducted in Anglo-Saxon countries where searches are carried out in English. Instead, we propose to carry out a pioneering study in Spain, where the language used the most in internet searches is Spanish. This represents a field of study that has been addressed very little in previous research. We have considered two work objectives:

1. To identify, through Google Trends, the annual time points from 2015 to 2019 with the highest Google search traffic for the terms “autism” and “Asperger” in Spain.
2. To identify the social and/or cultural events related to ASD that took place during the weeks with the highest number of searches on Google.

These objectives are relevant for two fundamental reasons: first, they can provide new information about infodemiological aspects related to autism in a country where this type of study has not previously been carried out. Additionally, the results of the present study may be useful to assess the real impact that recent educational and social inclusion policies in Spain have had on the search behavior of Spanish internet users.

## 2. Materials and Methods

To identify the periods of each year when there is a higher volume of Google queries related to autism or Asperger’s, the public database Google Trends was used; to identify possible social events related to the increase in the search volume, information from the MyNews database and from the trendinalia.com platform was used.

### 2.1. Google Trends

Google Trends presents relative search volume data in a standardized format. This database does not provide information about the absolute number of searches for a term, but it does provide information about the evolution of the number of searches for a specific term in a specific period of time. It gives a value of 100 to the specific date when there is a higher volume of searches in a given period, and from these data, proportional values are given according to the searches carried out. If a date receives a value of 50, it means that at that specific moment, 50% of the searches were performed compared to the date with a value of 100 in the same period of time.

Ten files in csv format were downloaded from Google Trends: five of them with the information related to Google searches for the term “Asperger” (one for each year from 2015 to 2019), and the other five with the information related to the searches for “autism”. In the files with the searches for the term “Asperger”, the value provided by Google Trends for the week of 18 February, coinciding with IAD, was found; in the files with the searches for the term “autism”, this same value was found for the week of April 2nd, coinciding with WAAD. In addition, for both types of files, the week with the highest volume of searches for each year was found. If this peak coincided only with the worldwide day, we looked for the next week with the highest search volume for both terms.

### 2.2. MyNews

MyNews is a press database that stores information from 1500 media outlets and has provided digital access to articles published in the Spanish press since 1996. It was used to search for news related to Asperger syndrome or autism in each of the identified weeks with the highest volume of searches on Google for the terms “Asperger” and “autism.” Therefore, 10 searches were carried out with the search term “Asperger” and 10 with the term “autism”. The searches found the number of publications in which our keywords (“Asperger” and “autism”) appear in any section of the article. Searches were limited to national, regional, or local media in Spain (except abroad). The total number of media covered by these parameters was 1349 and the percentage of news items published during the specific week analyzed was calculated for each result.

### 2.3. Trendinalia

Trendinalia is a database that stores the labels that have been TT worldwide in Twitter from 2013 to the present. For each day, Trendinalia gathers a list of the most popular topics, organized by the time they were a TT.

In order to identify the content of conversations in this social network, we searched in Trendinalia for the topics that had been TT on Twitter in Spain during the weeks with the highest number of searches (according to Google Trends results). A search for every single day during the 20 weeks with the highest volume of searches was carried out to identify conversations in which Asperger syndrome or autism was mentioned. To do this, the browser’s search option was chosen to find the expressions “Asper *” or “aut *”. Additionally, a qualitative review was performed, one by one, of all the TT for every single day searched. If the TT was unclear, a search for conversations on Twitter that used the tag at that moment was carried out, in order to discover the context and figure out the real meaning.

## 3. Results

**Figure 1:**
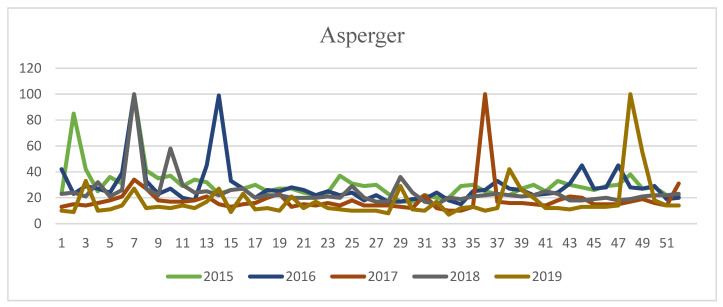
Evolution of annual searches in Google for the term “Asperger” (Spain, 2015–2019).

**Figure 2:**
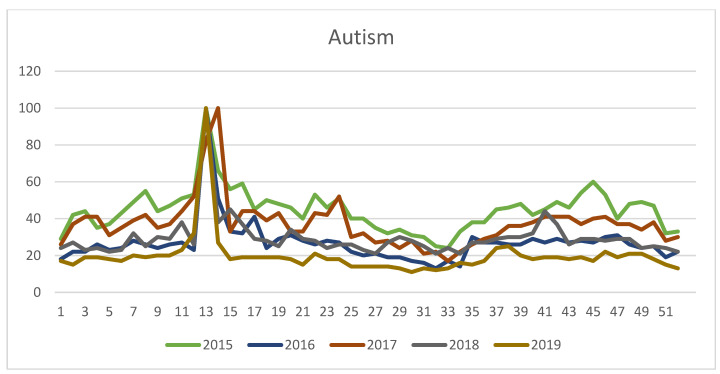
Evolution of annual searches on Google for the term “autism” (Spain, 2015–2019).

Figure 1 and Figure 2 show the evolution of the Google search volume from 1 January 2015 to 31 December 2019 for the terms “Asperger” and “autism”, respectively.

Figure 1 shows that search peaks are located in the seventh week of the year in 2015, 2016, and 2018, coinciding with International Asperger’s Day (in 2016 there is a double peak in the 7th and 17th weeks of the year); whereas in 2017 the peak is in the 39th week, and in 2019 in the 48th week.

Figure 2 shows that search peaks are located, for all the years analyzed, in the 13th or 14th weeks of the year, coinciding with the WAAD.

Table 2 and Table 3 gather three pieces of information for the terms “Asperger” and “autism”, respectively: the two weeks in which the highest volume of searches were carried out in each of the years between 2015 and 2019, the number of news items published in the Spanish press related to the corresponding keyword, and a possible explanation for the high number of searches during these weeks.

Table 4 and Table 5 show the TTs in Spain created during the two weeks with the highest volume of searches related to Asperger’s and autism for each year from 2015 to 2019; the position they occupied in the daily TT list; and the time they were TTs.

## 4. Discussion

In this study, our objectives were to determine at what times of the year the most Google searches about “Asperger” and “autism” were carried out in Spain from 2015 to 2020, and identify what social events may have caused this increase in internet users’ interest in locating information about ASD. After analyzing the searches in the years from 2015 to 2019, we found ten search peaks for each term: “Asperger” and “autism”. Generally, and for both diagnoses, these are stable, cyclical, stationary, and predictable peaks.

With regard to “Asperger” searches in Spain, in three of the years reviewed in this study (2015, 2016, and 2018), the weeks with the highest volume of searches in the Google Trends tool coincide with the celebration of IAD. In contrast, in 2017 and 2019, this event failed to trigger as many searches as other Asperger-related events that sparked higher search volumes. These events were a viral news story about a group of Argentine mothers who celebrated the expulsion of a child with Asperger’s from school and the United Nations Climate Summit Week, an event already identified as the reason for Google searches related to “Asperger” in the Anglo-Saxon context [17].

In the case of autism, for all the years reviewed in this study, the annual peaks of searches for this term on Google Trends coincide with WAAD, a result that is consistent with what was detected internationally in another study [25]. In fact, it seems that WAAD not only produces a sporadic increase in searches, but also that this increase has some durability over time. In 2015 and 2016, the week of the year with the highest volume of searches coincided with the celebration of WAAD; the second week (in terms of volume of searches) was the following week. This result suggests that WAAD has a longer impact than IAD on the increase in Google searches.

The other events that have increased the volume of searches on Google in Spain during the reviewed years are two viral news items (the inclusion of an autistic character in Sesame Street and the news about some parents who hid a camera in the backpack of their daughter with autism) and the promotion in Spain of the TV series “The Good Doctor” by its main actor, Freddie Highmore, who plays a young autistic doctor. The impact of these types of TV series and their promotions has also been identified in the Anglo-Saxon context as events potentially related to the increase in interest in autism on the internet [35]. Therefore, after analyzing the events that motivate internet users to search for “Asperger” and/or “autism” on Google in Spain, we discovered three types of circumstances: positive, negative, and ambivalent.

Among the positive circumstances, the analysis of the specific moments of the search peaks for both diagnoses in Spain demonstrated the success of international days, as occurs in other contexts [26]. This success is probably greater in the case of WAAD than for IAD because, in 2016, when WAAD was held, there was an upturn in searches for the term “Asperger” similar to the one produced by IAD. Likewise, with the promotion of the series “The Good Doctor” or the inclusion of a character with autism in Sesame Street, it was possible to make visible, give voice to, and create positive attitudes towards these two diagnoses [35,36].

On some occasions, the characteristics of the events that cause the increase in the volume of searches for Asperger and autism can also be negative [8,37], such as the news about the group of Argentine mothers who celebrated on WhatsApp the expulsion from school of a minor with Asperger’s in 2018, or the story of the parents who put a camera in the backpack of their daughter with autism to discover his harassment at school by teachers.

Finally, some sources that arouse interest in ASD have to do with phenomena that generate very different and mixed reactions. This would be the case of the screening of the film My name is Khan (a drama in which the main character has Asperger’s). This theme, apart from being close to becoming a cinematographic subgenre, can produce positive effects by publicizing the world of autism. It also causes obvious risks such as over dimensioning or under representing some characteristics of autism, generating a biased or stereotyped portrait of people with ASD [38]. This ambivalence in the effects of the cinematographic portrayal is not exclusive to ASD because it has also been identified for other diagnoses [39].

Similarly, Greta Thunberg could be considered another ambivalent phenomenon in ASD that creates high expectations, but with conflicting positions. She is a young activist with Asperger’s who has been named Person of the Year by Time magazine, but who in turn has been the subject of harsh criticism from certain internet users [16,17,18].

Regarding the volume of publication of news, the weeks with the highest number of searches for “Asperger” or “autism” on Google in Spain coincided with the publication of a large number of news stories about this subject (compared to the rest of the weeks of the year nationwide). If we assume that the total number of news items about ASD are published homogeneously throughout the year, each week 1.92% of the annual news should be published (it is the result of dividing 100% of the news by 52 weeks). However, an analysis of the data in Table 2 and Table 3 shows that the percentage of news published during the weeks with the highest volume of internet searches nationwide is significantly higher than this hypothetical 1.92%. Specifically, the percentage ranged between 2.1% and 10.3% in the case of the term “Asperger”, and between 2.2% and 9.7% in the case of the term “autism”. These results suggest that publications in the written press may be related to the increase in internet searches.

The results also show that the highest volume of searches is closely related to the TT on Twitter. In 9 of the 10 weeks with the highest volume of searches for “Asperger” and 9 of the 10 highest weeks for “autism”, there was at least one TT on Twitter related to these diagnoses. These results suggest the existence of links between social trends and web trends. In fact, these links have, according to previous studies [40], possible causal relationships. The clearest result of the present study occurred between 2 and 7 December in 2019, the week with the highest volume of searches of the year for the term “Asperger” (far from the next period with such a high impact). During this week, there were up to eleven TTs on Twitter related to Greta Thunberg, who reached the fifth and seventh position in the national TT list. With regard to autism, on 2 April 2019, there were five simultaneous TTs in Spain related to this diagnosis. The scope of autism can be considered greater if we look at the position it occupied on the TT list. In 2016 and 2017, the hashtag #DiaMundialAutismo reached the second position on the TT list, remaining at the center of the debate for 12 to 14 h. In 2019, seven of the 10 TT on autism had to do with WAAD. It is remarkable that the other three TT arise from the promotion of “The Good Doctor” series and the interviews with the actor.

Finally, it should be taken into account that being a TT depends on many circumstances: other TTs it competes with that day, if the TT is for a positive event or one that dirties the image of ASD, if the TT arises from an organized campaign (WAAD) or from a random event such as the influence of an actor (Freddie Highmore), or whether the hashtag used to tweet about the same event on the same day is unanimous or there is diversity (for example, in 2019, autism occupied the 5th position in TT #DiaMundialAutismo, but it should be noted that there were four other hashtags referring to the same event: autismo, 22nd; AutismDay2019, 42nd; Día Mundial del Autismo, 75th; Trastorno del Espectro Autista, 278th and #diamundialdelautismo, 371st).

In the present study, it was possible to determine the peak moments in searches for ASD, as well as the possible events that caused them. However, a limitation of this work is that only quantitative data were used. This quantitative analysis opens up new future lines of research that could carry out a qualitative analysis of the contents of TT, news published in the media, and responses Google provides to searches related to ASD. Likewise, future studies should research what motivates internet users to search for Asperger syndrome and autism on Google, but using data from different Spanish regions or from other countries in the international context where this type of study has not yet been carried out.

## 5. Conclusions

Campaigns related to IAD and WAAD are crucial tools to awaken our awareness of ASD, and they play a key role in shaping the social perception of Asperger’s and autism. In these campaigns, social networks and the publication of news act as powerful speakers, multiplying the impact of the International Conference campaigns.

Apart from these campaigns, there are other social or cultural events that also play an important role in shaping the social perception of ASD. Some of these events contribute to the configuration of positive attitudes towards ASD (such as the inclusion of characters with autism in series like Sesame Street or The Good Doctor). However, unfortunately, there are also events that we can consider ambivalent, or even directly related to negative aspects (for example, the Argentine mothers who celebrated the expulsion of a child with Asperger’s or the harassment of a child with autism recorded by a camera hidden in her backpack). The way in which social networks and the media present these events is not neutral, but can respond to the interests of those who dominate these agents. The fact that numerous media coincide in reproducing and magnifying unfortunate news, in which people with ASD are treated unfairly, suggests a certain stigmatizing pattern that shows people with ASD as weak people, who must confront obstacles. On the one hand, this type of news can contribute to empathize with people with ASD. But on the other hand, it contributes to creating a biased portrait, oversizing situations that fortunately are rare, and, at the same time, silencing countless success stories of people with ASD who are good examples of successful situations in social inclusion processes. In short, the media and social networks contribute to building the social image of people with ASD, a key element for their social inclusion.

For this reason, it is necessary to continue to create manuals with recommendations and protocols of good practices (such as the one developed by a Spanish association of ASD [41]), so that the media do not make common mistakes that could cloud all the work and awareness-raising efforts carried out on international days. The path towards the inclusion of people with ASD depends on several very different kinds of factors. Many of them are difficult to identify and check. For years, the social model of disability [42] has tried to identify and remove the disabling barriers existing in society. In the current knowledge society, probably some new barriers are being created in social media. Therefore, it will be difficult to achieve social inclusion if we do not pay enough attention to the portrayal of autism in the internet, social networks, and the press because it is the key to the way society shapes a collective image of ASD: it is its letter of introduction.

In this line, it is essential that associations, as well as those responsible for developing public policies related to inclusion, and trainers and professionals who work with children with ASD know what information is included about ASD in the net. In this sense, it will be possible to modulate the awareness-raising actions carried out by the associations, in addition to banishing possible myths and/or false beliefs, being able to really make the inclusion principle effective.

## Figures and Tables

**Table 1:** Changes from DSM-IV-TR categorization to DSM-5: Asperger syndrome and autism spectrum disorder (ASD).

Classification According to DSM-IV-TR ^1^	Classification According to DSM-5 ^1^
Pervasive Developmental Disorders Autistic disorder Rett’s disorder Childhood disintegrative disorder Asperger’s Disorder	Autism spectrum disorder Level 1: requiring support Level 2: requiring substantial support Level 3: requiring very substantial support

^1^ DSM-IV-TR [1] and DSM-5 [2] are two editions of the Diagnostic and Statistical Manual of Mental Disorders where these kinds of diagnoses are described.

**Table 2:** Weeks with the highest number of searches for the term “Asperger” on Google, news related to Asperger’s (Spain, 2015–2019), and social events explaining the volume of searches ^1^.

Week	Google Trends	News That Week (% of the Year)	Social Event
11/01/2015 to 17/01/2015	85	73 (2.1%)	Broadcast general TV. Telecinco Spanish channel film: “My name is Khan” in prime time (Saturday 17/01/2015)
15/02/2015 to 21//02/2015	100	353 (10.3%)	IAD ^2^
14/02/2016 to 20/02/2016	100	189 (6.1%)	IAD
03/04/2016 to 09/04/2016	99	89 (2.9%)	WAAD ^3^
12/02/2017 to 18/02/2017	34	224 (5.7%)	IAD
03/09/2017 to 09/09/2017	100	222 (5.6%)	Viral news: Argentine mothers celebrate on WhatsApp the expulsion from the school of a child with Asperger syndrome
18/02/2018 to 24/02/2018	100	250 (6.6%)	IAD
11/03/2018 to 17/03/2018	58	204 (5.4%)	Viral news: three students spray bleach on a child with Asperger
17/02/2019 to 23/02/2019	27	279 (5.4%)	IAD
01/12/2019 to 07/12/2019	100	384 (7.4%)	From December 2nd to 13th in Madrid the Climate Change Conference (COP 25) took place

^1^ Own elaboration based on Google Trends and MyNews data. Google Trends: search data for “Asperger” in the analyzed week provided by Google Trends; News this week (% of the year): number of news items containing the term “Asperger” during the analyzed week in any national, regional, or local media in Spain. In parentheses, we find the percentage that this news represents of the total news containing “Asperger” for the whole year; Social event: social events in the week related to the number of searches for the term “Asperger”. ^2^ “IAD” are the initials of International Asperger’s Day. ^3^ “WAAD” are the initials of World Autism Awareness Day.

**Table 3:** Weeks with the highest number of searches for the term “autism” on Google, news related to autism (Spain, 2015–2019), and social event explaining the volume of searches ^1^.

Week	Google Trends	News That Week (% of the Year)	Social Event
29/03/2015 to 4/04/2015	100	718 (4.7%)	WAAD
05/04/2015 to 17/04/2015	66	403 (2.6%)	WAAD
27/03/2016 to 02/04/2016	100	1740 (9.7%)	WAAD
03/04/2016 to 09/04/2016	51	823 (4.6%)	WAAD
19/03/2017 to 25/03/2017	52	510 (2.6%)	Viral news: Sesame Street incorporates a character with autism
02/04/2017 to 08/04/2017	100	1046 (5.3%)	WAAD
01/04/2018 to 07/04/2018	100	1385 (6.7%)	WAAD
15/04/2018 to 21/04/2018	45	771 (3.7%)	Viral news: parents put a hidden recorder inside the backpack of their child with ASD and discover harassment by the teachers
24/03/2019 to 30/03/2019	32	454 (2.2%)	Freddie Highmore (actor who plays a doctor with ASD in the TV series “The Good Doctor”) visits Madrid to promote his work in events and interviews on Spanish TV programs
31/03/2019 to 06/04/2019	100	1593 (7.8%)	WAAD

^1^ Own elaboration based on Google Trends and MyNews data. Google Trends: search data for “autism” in the analyzed week provided by Google Trends; News this week (% of the year): number of news items containing the term “autism” during the analyzed week in any national, regional, or local media in Spain. In parentheses, we find the percentage that this news represents of the total news containing “autism” for the whole year; Social event: social events in the week related to the number of searches for the term “autism”.

**Table 4:** TT in Spain during the weeks with the highest volume of searches for “Asperger” on Google ^1^.

Date	Trending Topic (TT)	Position	Duration (H:MM)
17/01/2015	Khan	14	4:40
	#MinombreesKhan	48	0:40
17/02/2016	#mesaAsperger	247	1:25
18/02/2016	#Asperger	41	6:35
	SerAsperger	252	1:30
19/02/2016	#Asperger	189	2:05
03/04/2016	#DiaMundialAutismo	18	7:15
	LNTAutismo	54	4:25
18/02/2017	#DíaInternacionalAsperger	7	10:25
03/09/2017	Asperger	184	1:55
05/09/2017	#Asperger	187	2:05
	Asperger	230	1:40
18/02/2018	#DíaInternacionalAsperger	24	8:15
	#Asperger	330	0:20
	Asperger	347	0:15
19/02/2018	#DíaInternacionalAsperger	38	6:10
15/03/2018	Asperger	98	3:50
19/02/2019	#DíaInternacionalAsperger	55	6:25
	Asperger	345	0:20
02/12/2019	#Greta Thunberg	18	9:25
	Greta	101	4:10
03/12/2019	Greta	38	7:05
	#Greta Thunberg	179	2:25
	Greta Thunberg	248	1:35
04/12/2019	#Greta Thunberg	5	10:50
	Greta	181	2:25
05/12/2019	Greta	21	8:40
06/12/2019	Greta Thunberg	154	2:30
	Greta	274	0:50
07/12/2019	Greta	7	11:45

^1^ Own elaboration based on Trendinalia data.

**Table 5:** TT in Spain during the weeks with the highest volume of searches for “autism” on Google ^1.^

Date	TT	Position	Duration (H:MM)
02/04/2015	#DiaMundialAutismo	6	9:10
01/04/2016	#DiaMundialAutismo	87	4:30
02/04/2016	#DiaMundialAutismo	2	14:35
	#AutismDay2016	348	0:10
03/04/2016	#DiaMundialAutismo	18	7:15
	#LNTAutismo	54	4:25
02/04/2017	#DiaMundialAutismo	2	12:30
03/04/2017	#DiaMundialAutismo	12	9:10
02/03/2017	Barrio Sésamo	229	1:15
02/04/2018	#DiaMundialAutismo	15	9:45
	#DiaInternacionaldelAutismo	72	4:50
	#diamundialdelutismo	166	2:15
03/04/2018	#DiaMundialAutismo	17	6:35
02/04/2019	#DiaMundialAutismo	5	10:25
	autismo	22	8:05
	AutismDay2019	42	7:10
	Día Mundial del Autismo	75	5:20
	Trastorno del Espectro Autista	278	1:10
	#diamundialdelautismo	371	0:10
03/04/2019	#DiaMundialAutismo	32	7:05
	autismo	37	6:35
04/04/2019	#DiaMundialAutismo	95	4:25
25/03/2019	#GoodDoctorAXN	353	0:15
	#FreddieEnMadrid	346	0:20
27/03/2019	Freddie Highmore	252	1:35

^1^ Own elaboration based on Trendinalia data.
